# Short-term outcomes of robotic versus laparoscopic TAPP for inguinal hernia repair: a systematic review, meta-analysis, and GRADE assessment

**DOI:** 10.1007/s11701-026-03335-3

**Published:** 2026-04-06

**Authors:** Mohamed H. Zidan, Mohamed AlSayed, Mario Maged, Hashem Altabbaa, Abdalla M. Hadhoud, Mahmoud Ashraf Hussein, Abedalrahman Yazan Aljarrah, Mohamed Hany, Ahmed Amgad, Mahmoud Albashier

**Affiliations:** 1 Alexandria University, Alexandria, Egypt; 2 The Research Papyrus Lab, Alexandria, Egypt; 3 Madina Bariatric Center, Madina Women’s Hospital, Alexandria, Egypt; 4 Department of Upper Gastrointestinal and Liver Surgery, Alexandria Main University Hospital, Alexandria University, Unit 2, Alexandria, Egypt; 5 Department of Surgery, Faculty of Medicine, Alexandria University, Alexandria, Egypt; 6 Faculty of Medicine, Cairo University, Cairo, Egypt; 7 Al-Basheer-Hospital, Amman, Jordan; 8 Department of Surgery, University of Cincinnati, Cincinnati, OH USA; 9 Faculty of Medicine, Capital University, Cairo, Egypt; 10 Princess Basma Hospital, Ministry of Health, Irbid, Jordan; 11 Medical Research Institute, Alexandria University, Alexandria, Egypt; 12 Faculty of Medicine, Damietta University, New Damietta, Egypt

**Keywords:** Robotic surgery, Laparoscopic surgery, Transabdominal preperitoneal repair, Inguinal hernia, R-TAPP, L-TAPP, Systematic review, Meta-analysis, Surgical outcomes, Cost analysis, Surgeon ergonomics

## Abstract

Robotic transabdominal preperitoneal (R-TAPP) repair has gained increasing adoption in inguinal hernia surgery, yet its short-term clinical value relative to conventional laparoscopic TAPP (L-TAPP) remains debated. Although robotic platforms offer technical and ergonomic advantages, whether these translate into measurable patient-centered benefits is uncertain. This systematic review and meta-analysis aimed to provide an updated comparative evaluation of short-term safety, efficacy, economic impact, and surgeon workload between R-TAPP and L-TAPP. A systematic review adhering to PRISMA 2020 standards was conducted across five major databases from inception through September 2025. Randomized controlled trials and comparative observational studies involving adult patients undergoing R-TAPP or L-TAPP were included. The primary endpoint was chronic postoperative inguinal pain. Secondary outcomes comprised operative duration, estimated blood loss, length of hospital stay, postoperative complications, recurrence, readmission, reoperation, urinary outcomes, surgical site infection, procedural costs, and surgeon workload assessed using the NASA Task Load Index (NASA-TLX). Risk of bias was evaluated using RoB 2 and ROBINS-I tools, and pooled analyses were performed using random-effects models. Fourteen independent studies (3 randomized trials and 11 cohort studies) encompassing 5,520 patients met the inclusion criteria. The incidence of chronic postoperative pain (*n* = 616) did not differ significantly between approaches (RR 0.58; 95% CI 0.30–1.12; *p* = 0.11; I² = 0%). Robotic repair was associated with prolonged operative time (MD 10.93 min; 95% CI 4.18–17.67; *p* = 0.002). No statistically significant differences were observed in blood loss, hospital stay, overall morbidity, recurrence, readmission, reoperation, urinary retention, urinary tract infection, or surgical site infection. While total NASA-TLX scores were comparable, robotic surgery significantly reduced the surgeon’s physical workload. Procedural costs were consistently higher in the robotic cohort. R-TAPP provides short-term clinical outcomes comparable to L-TAPP but is associated with longer operative times and increased direct costs. Its principal advantage appears to lie in improved surgeon ergonomics rather than superior patient outcomes. Robotic repair may therefore be most appropriate in complex cases or high-volume centers with established expertise, whereas laparoscopic TAPP remains a cost-efficient standard for routine inguinal hernia repair.

**Supplementary Information:**

The online version contains supplementary material available at 10.1007/s11701-026-03335-3.

## Introduction

Inguinal hernia repair remains among the most frequently performed procedures worldwide [1, 2], with contemporary guidelines emphasizing durable repair and the prevention of chronic postoperative inguinodynia. Minimally invasive approaches such as transabdominal preperitoneal (TAPP) repair have demonstrated recurrence rates comparable to open surgery while offering improved recovery profiles [3]. Parallel to this evolution, robotic-assisted platforms have seen rapid adoption across healthcare systems, prompting renewed scrutiny of whether technological enhancements translate into measurable clinical benefit [4].

Robotic TAPP (R-TAPP) offers ergonomic advantages like wristed instrumentation and three-dimensional visualization, but these features alone do not guarantee better short-term patient outcomes and must be weighed against resource implications [5, 6]. Ergonomic considerations are significant, as reviews suggest robotic platforms may reduce surgeon fatigue in comparison to laparoscopy, often assessed through tools like the NASA-TLX [7, 8].

However, three issues hinder meaningful comparisons: first, variations in surgeon experience and learning curves can affect estimates of efficiency and outcomes [9, 10]. Second, economic evaluations are inconsistent due to varying definitions of “cost,” including factors like amortization and operating room time [10, 11]. Third, inconsistencies in capturing surgeon-centered outcomes limit the assessment of the ergonomic benefits of robotic systems [12].

In light of these considerations, we performed a systematic review and meta-analysis in accordance with PRISMA guidelines to evaluate studies directly comparing robotic and laparoscopic TAPP (L-TAPP) repair in adult patients. The principal endpoints of interest were operative duration and chronic postoperative inguinal pain. Secondary endpoints encompassed perioperative metrics, short-term morbidity, hernia recurrence, measures of healthcare resource utilization, procedural costs, and surgeon-reported workload when such data were available.

## Method

### Study protocol and registration

This review was designed and reported in accordance with PRISMA 2020 standards and aligned with methodological guidance from the Cochrane Handbook for Systematic Reviews of Interventions [13, 14]. The protocol was prospectively registered in PROSPERO (CRD420251168493), ensuring transparency of eligibility criteria, outcome definitions, and analytical strategy.

#### Eligibility criteria

Studies were considered eligible if they involved adult patients undergoing inguinal hernia repair and provided a head-to-head comparison between R-TAPP and conventional L-TAPP. Both randomized controlled trials and comparative observational studies were deemed appropriate for inclusion. Publications were excluded if they lacked a comparator group, were review articles or narrative reports, consisted solely of conference abstracts without complete datasets, involved pediatric populations, relied on historical controls, were not peer-reviewed, were unavailable in full text, or were published in a language other than English.

The principal endpoint of interest was chronic postoperative groin pain (inguinodynia), selected as a clinically meaningful patient-centered outcome. Chronic postoperative pain was chosen as the primary endpoint because it is a crucial patient-centered outcome after inguinal hernia repair. Additional endpoints included perioperative metrics and postoperative events such as operative duration, hospitalization length, intraoperative blood loss, procedural cost, seroma, hematoma, hernia recurrence, readmission, reintervention, urinary retention, urinary tract infection, surgical site infection, overall complication rates, and complication severity per the Clavien–Dindo grading system.

### Information sources and search strategy

A comprehensive literature search was performed across MEDLINE (accessed via PubMed), Embase, Scopus, the Web of Science Core Collection, and the Cochrane Central Register of Controlled Trials (CENTRAL) from database inception through September 12, 2025. To ensure thorough coverage, additional sources were explored, including ClinicalTrials.gov and Google Scholar Labs. Reference lists of all included articles and pertinent review publications were also manually screened to identify any studies not captured through the electronic search. Full strategies (MeSH/keywords, Boolean logic, and limits) are available in Electronic Supplementary Material (ESM) 1; core concepts include inguinal hernia, TAPP, and robotic/laparoscopic approaches. Duplicates were removed in EndNote 20, and screening was conducted in Rayyan. Two reviewers independently screened records and full texts, with adjudication by a third reviewer. Search reporting followed PRISMA 2020 and PRISMA-S [14, 15].

### Data extraction

Data collection was conducted using a standardized spreadsheet developed in Microsoft Excel and structured into three predefined domains. The first domain documented core study-level information, including author identification, geographic setting, sample size, mesh fixation technique, defect closure approach, materials utilized, and duration of follow-up. The second domain recorded baseline patient characteristics, such as age, sex distribution, body mass index, presence of diabetes, history of recurrent hernia, and smoking status. The third domain comprised all prespecified clinical endpoints relevant to the assessment of procedural efficacy and postoperative safety.

Two reviewers independently extracted data and resolved discrepancies with a senior author if needed. Incomplete or unclear data were reassessed by a third reviewer. We compared study characteristics to identify overlapping publications, checking names, registration numbers, authors, recruitment periods, and sample sizes. Multiple reports with similar characteristics were cross-validated to confirm they originated from the same cohort, ensuring that only the most comprehensive dataset was included in the meta-analysis.

### Risk of bias

Methodological quality was appraised using validated risk-of-bias instruments appropriate to the study design. Randomized controlled trials were independently assessed by two reviewers using the Cochrane Risk of Bias 2 (RoB 2) framework, which evaluates potential concerns across domains including the randomization process, deviations from intended interventions, completeness of outcome data, outcome measurement, and selective reporting [16]. For non-randomized comparative studies, bias was examined using the ROBINS-I tool, encompassing domains related to confounding, participant selection, classification of interventions, deviations from protocol, missing data, outcome assessment, and reporting bias [17]. Visual summaries of bias assessments were generated using the RobVis platform [18]. Discrepancies between reviewers were adjudicated through consultation with a senior investigator.

### Cohort De-duplication and verification of trial independence

To minimize bias from overlapping populations in our study, we developed the Cohort De-duplication and Verification of Trial Independence (CDVTI) method. This approach combined co-authorship network analysis with reviews of institutional affiliations and manual checks of sample sizes, follow-up durations, and baseline characteristics. Using the ggraph package in R Studio (version 4.3.3), we created co-authorship graphs to visualize collaboration patterns among studies comparing R-TAPP and L-TAPP.

These graphs helped identify research groups that published trials on similar patient populations. We further verified potential duplications by cross-referencing institutional affiliations, recruitment timelines, and trial characteristics. Trials with different titles or abbreviated authors were analyzed to ascertain cohort redundancy, retaining only the most comprehensive report.

The CDVTI method ensured that each patient population was counted only once in the meta-analysis, preserving cohort independence and reducing the risk of effect size overestimation. The steps in this process are shown in ESM 2, Figure S1.

### Certainty of evidence (GRADE)

The certainty of evidence for each outcome was evaluated independently by two reviewers in accordance with the GRADE framework. Domains considered in this assessment included risk of bias, inconsistency of results, indirectness of evidence, imprecision of estimates, and potential publication bias. Based on these criteria, the overall certainty was categorized into four levels: high, moderate, low, or very low [19, 20]. A corresponding evidence summary table was generated using GRADEpro software (version 3.6.1).

### Statistical analysis

Quantitative synthesis was performed using Review Manager (RevMan), version 5.4. Continuous variables were summarized using pooled mean differences with corresponding 95% confidence intervals, whereas binary outcomes were combined as risk ratios with 95% confidence intervals employing the Mantel–Haenszel approach. Anticipating variability in study populations, operative techniques, and methodological design, a random-effects model was adopted for all meta-analyses. Between-study heterogeneity was evaluated using the chi-square test (with statistical significance set at *p* < 0.10) in conjunction with the I² statistic to quantify inconsistency. To assess the robustness of pooled estimates, leave-one-out sensitivity analyses were conducted for principal endpoints. Subgroup analyses were undertaken only when an adequate number of studies were available and when a biologically or clinically plausible rationale justified stratification; these analyses were interpreted as exploratory. For outcomes supported by ten or more studies, potential small-study effects were visually examined using funnel plots, with Egger’s regression test applied as a complementary statistical assessment [21]. All outcomes were reported in accordance with PRISMA 2020 recommendations for systematic reviews and meta-analyses.

## Results

### Study selection

The literature search yielded 2,955 records across all databases. Following duplicate removal (*n* = 1,565), 1,390 unique citations remained for screening. Title and abstract review excluded 1,110 records that did not meet predefined eligibility criteria. The remaining 280 articles underwent full-text evaluation, resulting in 16 comparative studies deemed potentially eligible. These comprised 11 observational cohort studies [22–32] and 5 reports of randomized trials [33–37], which were subsequently assessed for cohort independence prior to quantitative synthesis.

#### Authorship network and dataset independence

Cross-Dataset Verification and Trial Integrity (CDVTI) was applied to all potentially eligible comparative studies to identify overlapping trial populations and ensure statistical independence. Before CDVTI adjudication, 16 publications were considered eligible. Following systematic cross-verification, two randomized trial reports were identified as non-independent publications derived from overlapping trial cohorts and were excluded in accordance with prespecified hierarchical criteria (ESM 2. Table S1). Specifically, Prabhu et al. (2020) [36] was retained as the index randomized trial, while Miller et al. (2023) [35] was excluded as a longer-term follow-up of the same cohort. Similarly, Valorenzos et al. (2025) [37] was included as the primary randomized trial report, whereas Arunthavanathan et al. (2025) [33] was excluded as a derivative analysis from the same dataset.

To complement CDVTI adjudication, an authorship network analysis was performed to assess investigator overlap across included studies. Network visualization demonstrated distinct authorship clusters largely corresponding to geographically and institutionally cohesive research groups, without evidence of unrecognized cohort duplication among observational studies (ESM 2. Figure S2; Table S2).

**Fig. 1:**
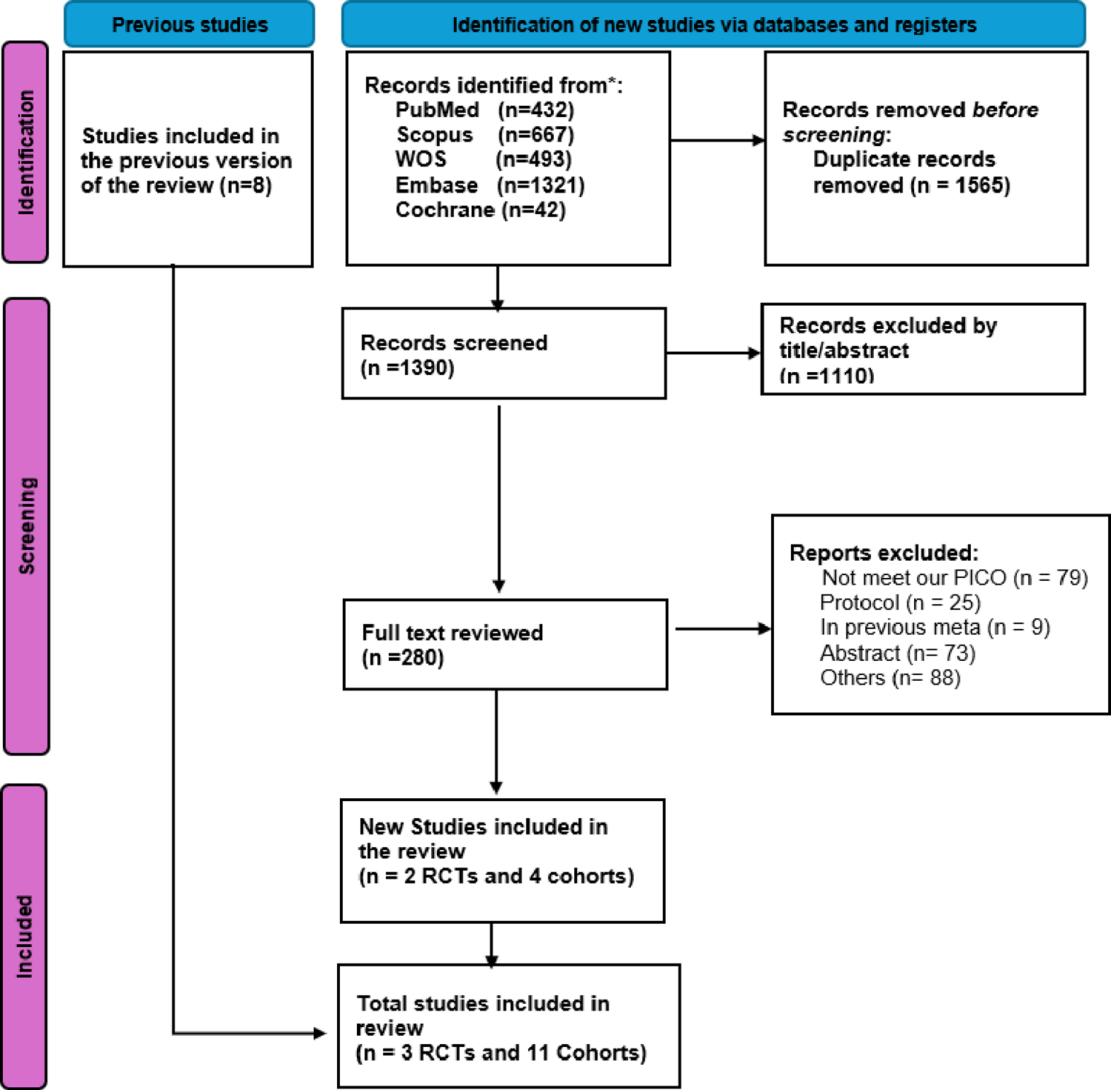
PRISMA 2020 flow diagram illustrating the study selection process for comparative studies of robotic versus laparoscopic transabdominal preperitoneal (TAPP) inguinal hernia repair. The diagram details record identification from databases and supplementary sources, duplicate removal, screening, full-text eligibility assessment, Cross-Dataset Verification and Trial Integrity (CDVTI) adjudication, and final inclusion in the quantitative synthesis

**Fig. 2:**
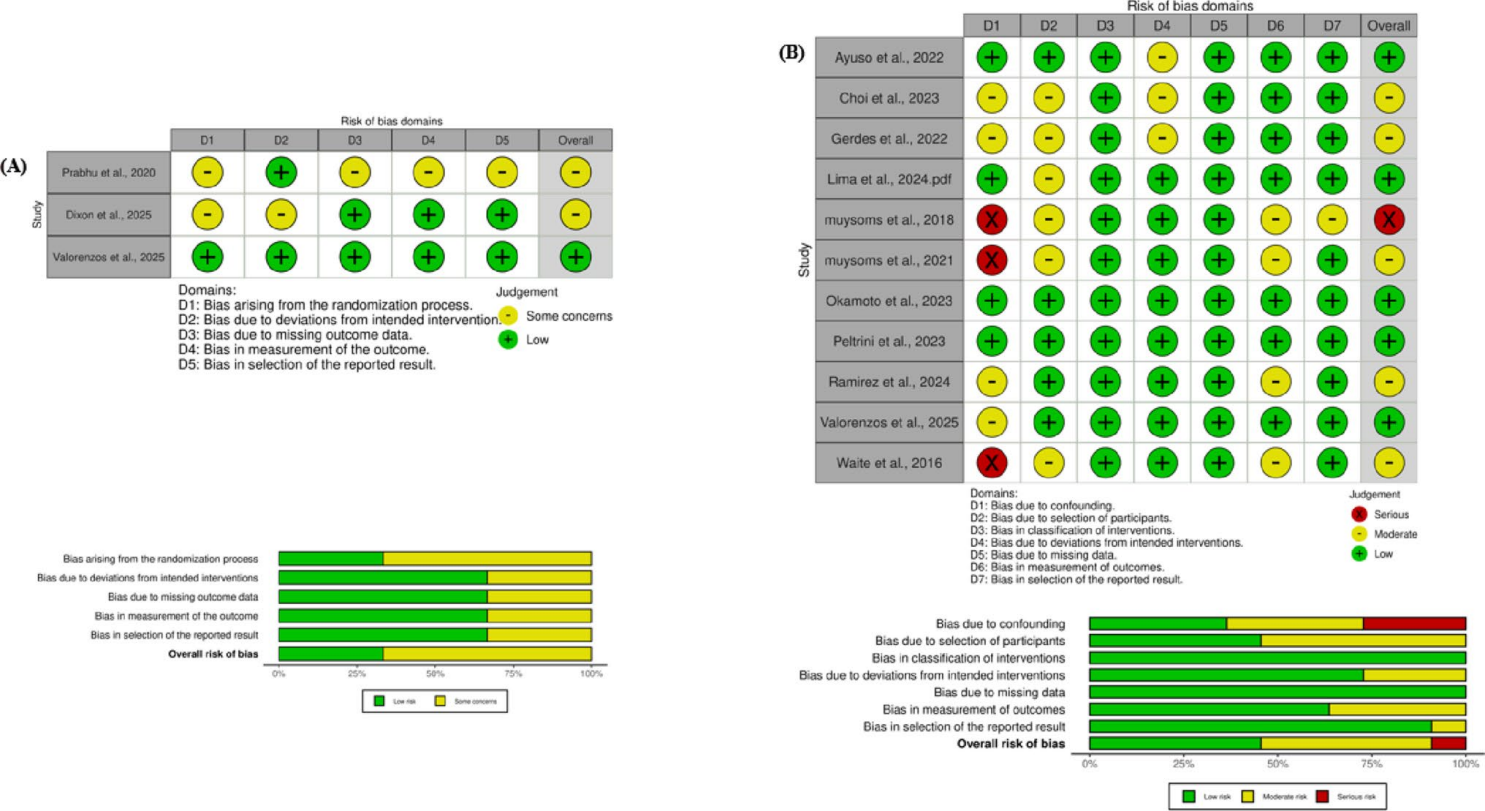
Risk of bias assessment of included studies. **(A)** Risk of bias in randomized controlled trials evaluated using the Cochrane Risk of Bias 2 (RoB 2) tool. **(B)** Risk of bias in non-randomized comparative studies assessed using the ROBINS-I tool. Risk-of-bias visualizations were generated using the *robvis* package

After application of the CDVTI framework to ensure dataset independence, 14 studies met the criteria for inclusion in the final meta-analysis. These consisted of 11 observational cohort investigations [22–32] and three randomized controlled trials [34, 36, 37]. A detailed overview of the identification, screening, and eligibility process is presented in the PRISMA flowchart (Figs. 1, 2).

### Study characteristics, bibliography, and demographics

Across the 14 eligible studies, a total of 5,520 patients were analyzed, with 2,692 individuals (48.76%) undergoing laparoscopic TAPP and 2,828 patients (51.24%) receiving robotic TAPP repair. The study populations were predominantly composed of male patients in the middle to older adult age groups, with reported mean ages generally spanning from the mid-50s to mid-60s across included cohorts.

The included studies spanned multiple geographic regions, predominantly Europe, followed by North America and East Asia, reflecting heterogeneous stages of robotic platform adoption. Earlier studies (2016–2018) largely represented initial robotic implementation and learning-curve phases, whereas more recent studies (2023–2025) increasingly employed randomized or propensity-matched designs and incorporated surgeon-centered outcomes, cost analyses, and standardized complication reporting.

Across studies, permanent synthetic meshes were universally used. Robotic TAPP repairs predominantly utilized self-fixating polyester or contoured polypropylene meshes, enabling reduced reliance on mechanical fixation. Suture-based fixation or no fixation was more common in robotic cohorts, leveraging articulated instrumentation and enhanced intracorporeal suturing capability, whereas laparoscopic repairs more frequently employed tacks or glue.

Defect closure was performed more consistently in robotic TAPP, typically using barbed absorbable sutures, reflecting both ergonomic advantages and evolving robotic surgical technique. In contrast, laparoscopic defect closure was variably reported, particularly in earlier publications. A temporal trend toward routine defect closure in robotic repairs was observed in contemporary studies.

Follow-up durations ranged from short-term (1 month) to long-term (> 6 years). Overall, variations in mesh selection, fixation strategy, defect closure practice, and publication era highlight the technical maturation of robotic TAPP and represent key sources of clinical heterogeneity relevant to interpreting comparative robotic outcomes.

The baseline demographics are presented in Table 1, and the trial-level design, along with operative characteristics, is outlined in Table 2.

**Table 1:** Baseline demographic and anthropometric data at baseline across all included studies comparing Robotic TAPP and Laparoscopic TAPP, including age, BMI, sex, prevalence of diabetes, smoking, and recurrent inguinal hernia

NO	Study ID	Type of Repair	Number in each group	Age (Years) **Mean ± SD**	BMI (kg/m²) **Mean ± SD**	Male **N (%)**	Diabetes (%) **N (%)**	Smoking (%) **N (%)**	Recurrent Inguinal Hernia **N (%)**
1	Ayuso et al., 2022 [22]	L-TAPP	141	54.4 ± 15.5	27.1 ± 5.1	NR	11 (7.8)	36 (25.5)	28 (19.9)
		R-TAPP	141	58.6 ± 13.8	29.1 ± 21	NR	7 (5)	40 (28.4)	16 (11.4)
2	Choi et al., 2023 [23]	L-TAPP	50	64.4 ± 14.8	23.8 ± 2.9	49 (98)	9 (18)	15 (30)	5 (10)
		R-TAPP	50	54.4 ± 14	24.8 ± 3	50 (100)	5 (10)	19 (38)	3 (6)
3	Gerdes et al., 2022 [24]	L-TAPP	29	53 ± 15.25	25 ± 3.5	24 (82.75)	NR	NR	NR
		R-TAPP	29	62 ± 11.25	24 ± 2.25	27 (93.1)	NR	NR	NR
4	Lima et al., 2024 [26]	L-TAPP	1,598	60 ± 3.66	26 ± 0.83	1,439 (90)	95(6)	165(10)	NR
		R-TAPP	1,598	60 ± 3.83	26 ± 0.66	1,463 (92)	85(5)	147(9)	NR
5	Muysoms et al., 2018 [27]	L-TAPP	64	57.7 ± 12.6	24.4 ± 3	62 (98.5)	NR	NR	0(0)
		R-TAPP	49	58.8 ± 15.4	25 ± 3.04	48 (98)	NR	NR	2(4)
6	Muysoms et al., 2021 [28]	L-TAPP	272	60.3 ± 62.0	NR	237(87.1)	11 (4.0)	45 (16.6)	17 (6.3)
		R-TAPP	404	60.0 ± 61.7	NR	377(93 3)	25 (6.2)	56 (13.9)	32 (7.9)
7	Okamoto et al., 2023 [29]	L-TAPP	80	71 ± 2.25	22.8 ± 0.58	75 (94)	4 (5)	NR	NR
		R-TAPP	80	70 ± 2.33	23.1 ± 0.56	76 (95)	3 (3.8)	NR	NR
8	Peltrini et al., 2023 [30]	L-TAPP	80	56 ± 14	25.54 ± 2.71	71 (89)	NR	18 (22)	20(25)
		R-TAPP	40	56 ± 12	25.37 ± 3.28	35(88)	NR	12 (30)	10(25)
9	Ramirez et al., 2024 [25]	L-TAPP	78	62.38 ± 13.88	27.32 ± 4.09	19 (78.2)	NR	NR	33 (42.3)
		R-TAPP	20	66.1 ± 11.48	26.7 ± 3.16	19 (95)	NR	NR	10(50)
10	Valorenzos et al., 2024 [31]	L-TAPP	177	64 ± 3.5	25.13 ± 0.58	144 (81.4)	8 (4.5)	33 (19.5)	34 (19.2)
		R-TAPP	218	63 ± 3.83	25.17 ± 0.68	182 (83.5)	11 (5.0)	50 (23.9)	46 (21.1)
11	Waite et al., 2016 [32]	L-TAPP	24	57.5 ± 7.25	27.6 ± 3.05	24 (100)	NR	NR	0(0)
		R-TAPP	39	58.1 ± 14.75	27.5 ± 3.21	38 (97.4)	NR	NR	2(5)
12	Dixon et al., 2025 [34]	L-TAPP	20	57.6 ± 13.7	24.2 ± 3.5	18 (90)	NR	3 (15)	NR
		R-TAPP	39	59.9 ± 15.2	27.1 ± 3.6	37 (95)	NR	2 (51)	NR
13	Valorenzos et al.,2025 [37]	L-TAPP	65	66.0 ± 3.75	25.9 ± 1.07	53 (81.5)	3 (4.6)	15 (23.1)	7 (10.8)
		R-TAPP	74	61.5 ± 3.3	25.6 ± 0.866	62 (83.8)	2 (2.7)	17 (23.0)	7 (9.5)
14	Prabhu et al., 2020 [36]	L-TAPP	54	57.2 ± 13.3	26.9 ± 4.42	48 (88.9)	4 (7.41)	6 (11.3)	3 (5.56)
		R-TAPP	48	56.1 ± 14.1	24.9 ± 3.24	44 (91.6)	2 (4.17)	3 (6.25)	5 (10.6)

**Table 2:** Summary of study region, design, sample size, Type of mesh, Method of Mesh Fixation, Defect closure, Defect closure material and follow-up intervals for each included studies comparing Robotic TAPP and Laparoscopic TAPP inguinal hernia repair

Study ID	Country	Study design	Total sample size	Surgical approach	Type of mesh	Method of mesh fixation (dominant)	Defect closure	Defect closure material	Follow-up duration (Months)
Lima et al., 2024 [26]	USA	RCS	6,392	ROB (TAPP)	Permanent synthetic mesh	Suture	Yes	NR	12 months
				LAP (TAPP)	Permanent synthetic mesh	Tacks	Yes	NR	
Ayuso et al., 2022 [22]	USA	RCS	282	ROB (TAPP)	Synthetic polypropylene mesh (mostly 3D Max, midweight)	Suture	Yes	Permanent, non-absorbable suture	1 month
				LAP (TAPP)	Synthetic polypropylene mesh (mostly 3D Max, midweight)	Tacks	Yes	NR	
Gerdes et al., 2022 [24]	Switzerland	PCS	58	ROB (TAPP)	Synthetic polypropylene mesh (BARD 3D Lightmesh)	No fixation	Yes	Absorbable suture	1.5 months
				LAP (TAPP)	Synthetic polypropylene mesh (BARD 3D Lightmesh)	No fixation	Yes	Absorbable suture	
Choi et al., 2023 [23]	South Korea	RCS	100	ROB (TAPP)	Synthetic polypropylene mesh (3DMax™ Light Mesh, large size)	Tacks	Yes	Suture	NR
				LAP (TAPP)	Synthetic polypropylene mesh (3DMax™ Light Mesh, large size)	Tacks	Yes	NR	
Muysoms et al., 2021 [28]	Belgium	RCS	676	ROB (TAPP)	Self-gripping monofilament polyester mesh (Parietex ProGrip™)	Self-fixating mesh	Yes	Barbed absorbable suture	1 month
				LAP (TAPP)	Self-gripping monofilament polyester mesh (Parietex ProGrip™)	Self-fixating mesh	Yes	Barbed absorbable suture	
Muysoms et al., 2018 [27]	Belgium	PCCS	99	ROB (TAPP)	Self-gripping polyester mesh (ProGrip™)	Self-fixating mesh	Yes	Barbed absorbable suture	1 month
				LAP (TAPP)	Self-gripping polyester mesh (ProGrip™)	Self-fixating mesh	Yes	Barbed absorbable suture	
Waite et al., 2016 [33]	USA	RCS	63	ROB (TAPP)	Contoured polypropylene mesh (3D Max); minority self-adhering mesh (ProGrip)	Suture	Yes	Running barbed suture	NR
				LAP (TAPP)	Contoured polypropylene mesh (Bard 3D Max); minority self-adhering mesh (ProGrip)	Tacks	Yes	Absorbable tacks	
Valorenzos et al., 2024 [31]	Denmark	RCS	395	ROB (TAPP)	Self-fixating polyester mesh (ProGrip™)	Self-fixating mesh	Yes	Barbed absorbable suture	Median follow-up time was 2215 days (6.1 years)
				LAP (TAPP)	Mixed meshes (self-fixating and non-self-fixating synthetic meshes)	**Tacks or sutures** (surgeon preference)	Yes	Tacks or sutures	
Ramirez et al., 2024 [25]	Spain	RCS	98	ROB (TAPP)	Polypropylene mesh (3D MAX™)	Tacks ± glue	Yes	Absorbable barbed suture	12 months
				LAP (TAPP)	Polypropylene mesh (3D MAX™)	Tacks ± glue	Yes	Absorbable suture	
Peltrini et al., 2023 [30]	Italy	RCS	120	ROB (TAPP)	Mixed synthetic meshes (Ultrapro^®^, ProGrip^®^, polypropylene; contoured)	Suture	Yes	Barbed absorbable suture	Lap: 52 ± 14 months
				LAP (TAPP)	Mixed synthetic meshes (Ultrapro^®^, Parietex^®^, ProGrip^®^, polypropylene)	**Fibrin glue** (except ProGrip cases)	Yes	Barbed absorbable suture	Rob: 35 ± 8 months
Okamoto et al., 2023 [29]	Japan	RCS	160	ROB (TAPP)	Self-gripping mesh, 15 × 10 cm (Lap ProGrip™, polyester)	Self-fixating mesh	Yes	Running absorbable monofilament suture	NR
				LAP (TAPP)	Flat synthetic mesh	Tacks	Yes	Running absorbable monofilament suture	
Prabhu et al., 2020 [36]	USA	Multicenter RCT	102	ROB (TAPP)	Flat heavyweight polypropylene mesh	Suture	Yes	Running suture	1 month
				LAP (TAPP)	Flat heavyweight polypropylene mesh	Permanent tacks	Yes	NR	
Dixon et al., 2025 [34]	UK	Single-center RCT	60	ROB (TAPP)	NR	NR	NR	NR	0.5 (14 days)
				LAP (TAPP)	NR	NR	NR	NR	
Valorenzos et al.,2025 [37]	Denmark	Single-center RCT	139	ROB (TAPP)	Self-fixating polyester mesh (ProGrip™)	Self-fixating mesh	Yes	Barbed absorbable suture	3 months
				LAP (TAPP)	Self-fixating polyester mesh (ProGrip™)	Self-fixating mesh	Yes	Barbed absorbable suture	

RCS Retrospective cohort study, PCS Prospective cohort study, PCCS Prospective case-control study, RCT Randomized controlled trial, ROB (TAPP) Robotic transabdominal preperitoneal inguinal hernia repair, LAP (TAPP) Laproscopic transabdominal preperitoneal inguinal hernai repair, NR Not reported

### Risk of bias

The methodological rigor of the three included randomized controlled trials was appraised using the Cochrane Risk of Bias 2.0 framework, with graphical summaries generated through the robvis tool (Fig. 2A). Most trials were judged to present “some concerns,” primarily related to aspects of the randomization process and minor imbalances in baseline characteristics. Allocation concealment procedures were generally appropriate, and incomplete outcome data posed minimal concern given the low attrition rates reported. No indications of selective reporting were identified. Taken together, the randomized evidence was considered to reflect an overall low-to-moderate risk of bias.

The eleven observational cohort studies underwent evaluation using the ROBINS-I instrument, with visual representation also produced via robvis (Fig. 2B). Overall, the majority of studies demonstrated a favorable methodological profile. Risk was predominantly low across key domains, particularly regarding confounding control, participant selection, and outcome assessment. Occasional moderate concerns were observed, mainly in relation to intervention classification and deviations from intended treatment protocols. Two studies were judged to carry a serious risk of bias due to limitations in confounding adjustment and selection processes. Nevertheless, these concerns were isolated, and the collective body of observational evidence was deemed methodologically sound, with most domains consistently rated as low risk.

### Certainty of evidence (GRADE)

According to the GRADE framework, the certainty of evidence was high for haematoma, recurrence, and procedural cost outcomes. Moderate-certainty evidence was identified for blood loss, length of hospital stay, seroma, readmission, urinary retention, complications classified by the Clavien–Dindo system, surgical site infection, and chronic postoperative inguinal pain (inguinodynia). The certainty of evidence was low for operative time, total NASA Task Load Index (NASA-TLX), overall postoperative complication rate, reoperation, and urinary tract infection. Detailed GRADE assessments and evidence profiles for all outcomes are provided in ESM 3.

### Meta-analysis

Following cohort de-duplication, 14 independent studies were included in the quantitative synthesis [22–32, 34, 36, 37].

#### Chronic postoperative pain (Inguinodynia)

**Fig. 3:**
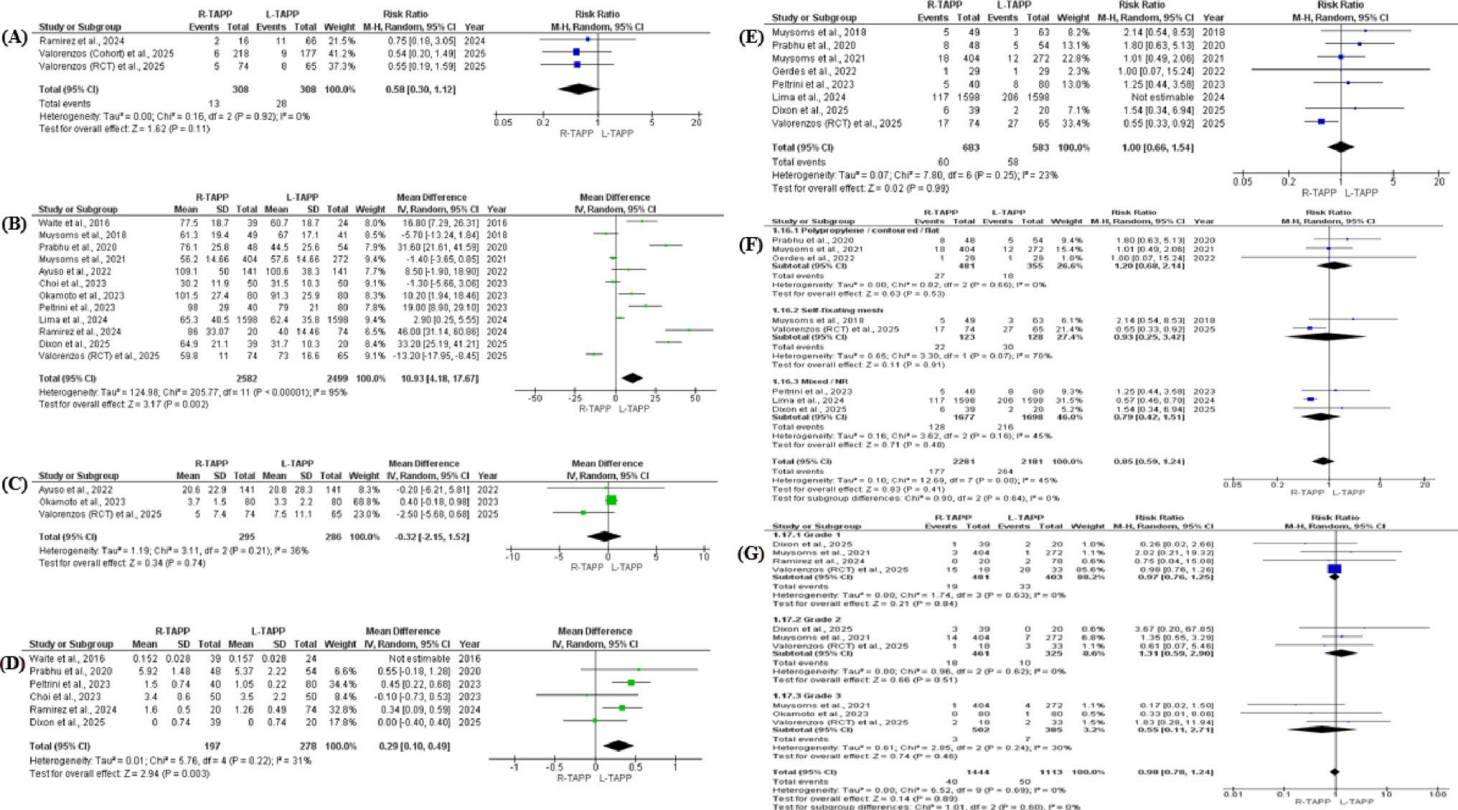
Forest plots comparing robotic versus laparoscopic TAPP inguinal hernia repair for primary, perioperative, and overall morbidity outcomes using random-effects meta-analysis. **(A)** Chronic postoperative inguinal pain (inguinodynia; risk ratio [RR]). **(B)** Operative time (minutes; mean difference [MD]). **(C)** Estimated blood loss (mL; MD). **(D)** Length of hospital stay (days; MD). **(E)** Overall postoperative complications (RR). **(F)** Overall complications stratified by dominant mesh type (polypropylene/contoured/flat, self-fixating, and mixed or not reported). **(G)** Postoperative complications stratified by Clavien–Dindo grade (RR). Effect estimates are presented with 95% confidence intervals (CIs); heterogeneity is reported using the I² statistic

Chronic groin pain following surgery was evaluated in three studies [25, 31, 37], comprising 616 patients in total, evenly distributed between robotic and laparoscopic groups (308 per cohort). Random-effects meta-analysis revealed no statistically significant difference in the incidence of chronic postoperative pain between R-TAPP and L-TAPP (RR 0.58; 95% CI 0.30–1.12; *p* = 0.11). The findings were highly consistent across studies, with no detectable heterogeneity (I² = 0%; *p* = 0.92) (Fig. 3A).

#### Operative time

Twelve studies [22, 23, 25–30, 32, 34, 36, 37] analysed the operative time in minutes between the two groups. A total of 2582 patients received R-TAPP, and 2499 patients underwent L-TAPP. Pooled analysis demonstrated a statistically significant prolongation of operative duration in the robotic cohort (MD 10.93 min; 95% CI 4.18–17.67), although heterogeneity was substantial (I² = 95%) (Fig. 3B), reflecting inter-study variability in surgeon experience, procedural workflow, and institutional adoption phase.

#### Blood loss

The estimated intraoperative blood loss was reported in three studies [22, 29, 37], comprising 295 patients in the robotic cohort and 286 in the laparoscopic cohort. Pooled analysis demonstrated no statistically significant difference between R-TAPP and L-TAPP (MD − 0.32; 95% CI − 2.15 to 1.52; *p* = 0.74). Between-study variability was low to moderate, as reflected by an I² value of 36% (Fig. 3C).

#### Length of hospital stay

Hospital length of stay was reported in six studies [23, 25, 30, 32, 34, 36], including 236 patients treated with R-TAPP and 302 with L-TAPP. The pooled mean difference indicated a small, non-significant increase in hospital stay associated with the robotic approach (MD 0.20 days; 95% CI − 0.04 to 0.43; *p* = 0.11). Substantial heterogeneity was present (I² = 79%), suggesting variability across study settings and perioperative protocols. Sensitivity analysis using a leave-one-out approach identified the study by Waite et al. (2016) [32] as a primary contributor to this heterogeneity. Exclusion of this study reduced inconsistency to 31% and shifted the pooled estimate to statistical significance (MD 0.29 days; 95% CI 0.10–0.49; *p* = 0.003) (Fig. 3D). This finding indicates that differences in sample size and operative methodology in the Waite study influenced the overall estimate. Nevertheless, the direction of effect remained consistent across analyses, suggesting that the general trend was stable, although the statistical significance depended on study inclusion.

#### Overall complication rate

Eight studies [24, 26–28, 30, 34, 36, 37] reported aggregate postoperative complication rates, representing a total of 4,462 patients (2,281 in the robotic group and 2,181 in the laparoscopic group). Meta-analytic pooling demonstrated no statistically significant difference between R-TAPP and L-TAPP (RR 0.85; 95% CI 0.59–1.24; *p* = 0.41). Moderate heterogeneity was observed (I² = 45%). Sensitivity analysis identified the study by Lima et al. (2024) as a contributor to between-study variability. Exclusion of this study reduced heterogeneity to 23%, while the pooled estimate remained non-significant (RR 1.00; 95% CI 0.66–1.54; *p* = 0.99) (Fig. 3E), supporting the robustness of the overall findings. Subgroup analyses based on predominant mesh type (polypropylene/contoured/flat, self-fixating, or mixed/not specified) did not reveal a significant interaction between mesh selection and surgical platform (χ² = 0.90; df = 2; *p* = 0.64; I² = 0%). Within each mesh category, complication rates were comparable between robotic and laparoscopic repair. The consistency of effect estimates across subgroups suggests that mesh type did not meaningfully alter the relative safety profile of the two approaches (Fig. 3F).

#### Complications according to Clavien–Dindo classification

Five studies [25, 28, 29, 34, 37] provided complication data categorized by Clavien–Dindo grade. When pooled, no statistically significant difference was identified between robotic and laparoscopic TAPP for overall graded complications (RR 0.98; 95% CI 0.78–1.24; *p* = 0.89), and no between-study heterogeneity was observed (I² = 0%).

Grade-specific analyses yielded similar findings. For minor complications (Grade I), the risk did not differ significantly between approaches (RR 0.97; 95% CI 0.76–1.25; *p* = 0.84; I² = 0%). Likewise, no significant differences were detected for Grade II events (RR 1.31; 95% CI 0.59–2.90; *p* = 0.51; I² = 0%) or Grade III complications (RR 0.55; 95% CI 0.11–2.71; *p* = 0.46). Across all severity categories, effect estimates remained statistically comparable (Fig. 3G).

#### Seroma

**Fig. 4:**
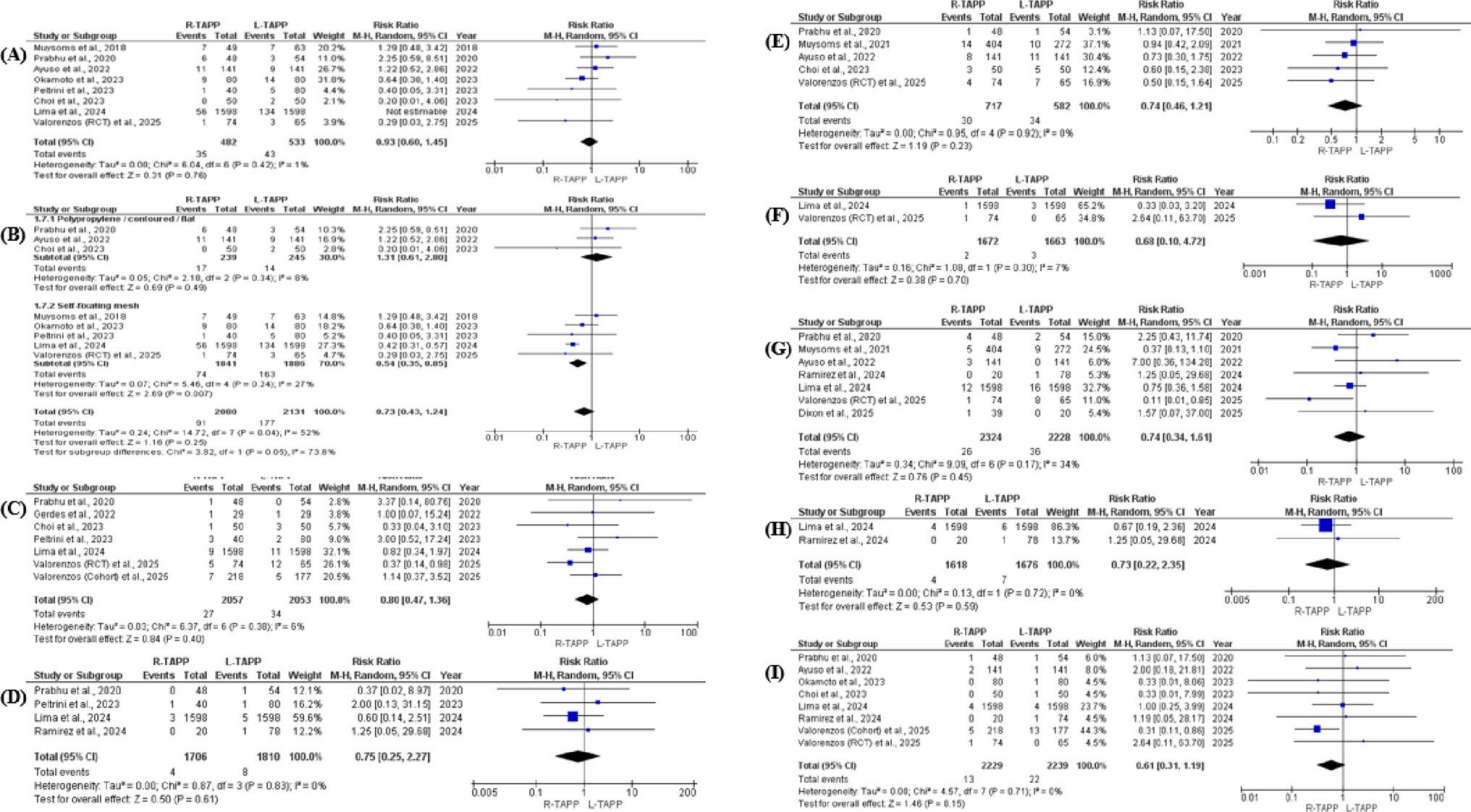
Forest plots comparing robotic versus laparoscopic TAPP inguinal hernia repair for specific postoperative morbidity, urinary outcomes, healthcare utilization, and durability outcomes using random-effects meta-analysis. **(A)** Seroma (overall analysis). **(B)** Seroma stratified by dominant mesh type (self-fixating vs. polypropylene/contoured). **(C)** Hematoma. **(D)** Surgical site infection. **(E)** Urinary retention. **(F)** Urinary tract infection. **(G)** Readmission. **(H)** Reoperation. **(I)** Recurrence. Effect estimates are presented as risk ratios (RRs) with 95% confidence intervals (CIs); heterogeneity is reported using the I² statistic

Eight studies [22, 23, 26, 27, 29, 30, 36, 37] reported postoperative seroma formation, including 4,211 patients overall (2,080 undergoing robotic repair and 2,131 undergoing laparoscopic repair). Random-effects meta-analysis demonstrated no statistically significant difference between R-TAPP and L-TAPP (RR 0.73; 95% CI 0.73–1.24; *p* = 0.25). Moderate between-study heterogeneity was observed (I² = 52%; *p* = 0.04). Sensitivity analysis using a leave-one-out approach identified the study by Lima et al. (2024) [26] as a primary contributor to heterogeneity. Exclusion of this study reduced inconsistency substantially (I² = 1%) while maintaining a non-significant pooled effect (RR 0.93; 95% CI 0.60–1.45; *p* = 0.76) (Fig. 4A), indicating overall stability of the findings. Exploratory subgroup analyses stratified by predominant mesh type suggested a potential interaction between mesh selection and surgical approach. In cohorts utilizing self-fixating meshes, robotic repair was associated with a significantly lower incidence of seroma compared with laparoscopy (RR 0.54; 95% CI 0.35–0.85; *p* = 0.007), with low heterogeneity (I² = 27%). Conversely, studies employing polypropylene or contoured meshes demonstrated no significant difference between techniques (RR 1.31; 95% CI 0.61–2.80; *p* = 0.49; I² = 8%). The test for subgroup interaction approached statistical significance (χ² = 3.82; df = 1; *p* = 0.05), suggesting that mesh characteristics may modulate the association between operative platform and seroma risk (Fig. 4B).

#### Haematoma

Seven studies [23, 24, 26, 30, 31, 36, 37] reported data on postoperative hematoma, representing a combined sample of 4,110 patients, with 2,057 in the robotic group and 2,053 in the laparoscopic group. Meta-analytic pooling under a random-effects framework showed no statistically significant difference between the two surgical approaches (RR 0.80; 95% CI 0.47–1.36; *p* = 0.40). The results were consistent across studies, as indicated by minimal heterogeneity (I² = 6%; *p* = 0.38) (Fig. 4C).

#### Surgical site infection

Postoperative surgical site infection was evaluated in four studies [25, 26, 30, 36], comprising 3,516 patients overall (1,706 in the robotic cohort and 1,810 in the laparoscopic cohort). Random-effects meta-analysis demonstrated no statistically significant difference between R-TAPP and L-TAPP (RR 0.75; 95% CI 0.25–2.27; *p* = 0.61). The pooled estimate showed complete consistency across studies, with no detectable heterogeneity (I² = 0%; *p* = 0.83) (Fig. 4D).

#### Urinary retention

Five studies [22, 23, 28, 36, 37] provided data on postoperative urinary retention, including 717 patients in the robotic group and 582 in the laparoscopic group. Meta-analytic synthesis demonstrated no statistically significant difference between the two techniques (RR 0.74; 95% CI 0.46–1.21; *p* = 0.23). The findings were highly consistent across studies, with no evidence of heterogeneity (I² = 0%; *p* = 0.92) (Fig. 4E).

#### Urinary tract infection

Postoperative urinary tract infection was reported in two studies [26, 37], comprising 3,335 patients overall (1,672 in the robotic group and 1,663 in the laparoscopic group). Random-effects pooling revealed no statistically significant difference between R-TAPP and L-TAPP (RR 0.68; 95% CI 0.10–4.72; *p* = 0.70). Measures of inconsistency indicated minimal variability across studies (I² = 7%; *p* = 0.30) (Fig. 4F). Collectively, these data suggest that the choice of surgical platform does not materially alter the risk of postoperative urinary tract infection, with findings remaining consistent across multicenter and geographically diverse cohorts.

#### Readmission rate

Seven comparative studies [22, 25, 26, 28, 34, 36, 37] provided data on postoperative readmissions, including 2,324 patients in the robotic cohort and 2,228 in the laparoscopic cohort. Meta-analytic pooling demonstrated no statistically significant difference between R-TAPP and L-TAPP (RR 0.74; 95% CI 0.34–1.61; *p* = 0.45). Between-study variability was low, with (I² = 34%) (Fig. 4G).

#### Re-operation rate

Reoperation rates were reported in two studies [25, 26], encompassing 1,618 patients treated with the robotic approach and 1,676 managed laparoscopically. The pooled estimate demonstrated no significant difference between techniques (RR 0.73; 95% CI 0.22–2.35; *p* = 0.59). There was no evidence of variability across studies, as indicated by (I² = 0%) (Fig. 4H).

#### Conversion to open surgery

Conversion to open repair was reported in five studies, including both cohort and randomized designs. Across these studies, conversion events were extremely rare. No conversions were reported in the robotic cohorts (0/223), whereas a single conversion occurred in the laparoscopic group (1/293). Given the very low event frequency, a formal meta-analysis was not considered statistically meaningful. Overall, both minimally invasive approaches demonstrated very low conversion rates when performed by experienced surgical teams.

#### Recurrence rates

Recurrence outcomes were reported in eight comparative studies [22, 23, 25, 26, 29, 31, 36, 37], including 4,468 patients overall (2,229 in the robotic group and 2,239 in the laparoscopic group). When pooled using a random-effects model, no statistically significant difference in hernia recurrence was observed between R-TAPP and L-TAPP (RR 0.61; 95% CI 0.31–1.19; *p* = 0.15). Measures of between-study variability indicated consistent findings across studies, with no detectable heterogeneity (I² = 0%; *p* = 0.71) (Fig. 4I).

#### Surgeon workload and ergonomics: total NASA-TLX

**Fig. 5:**
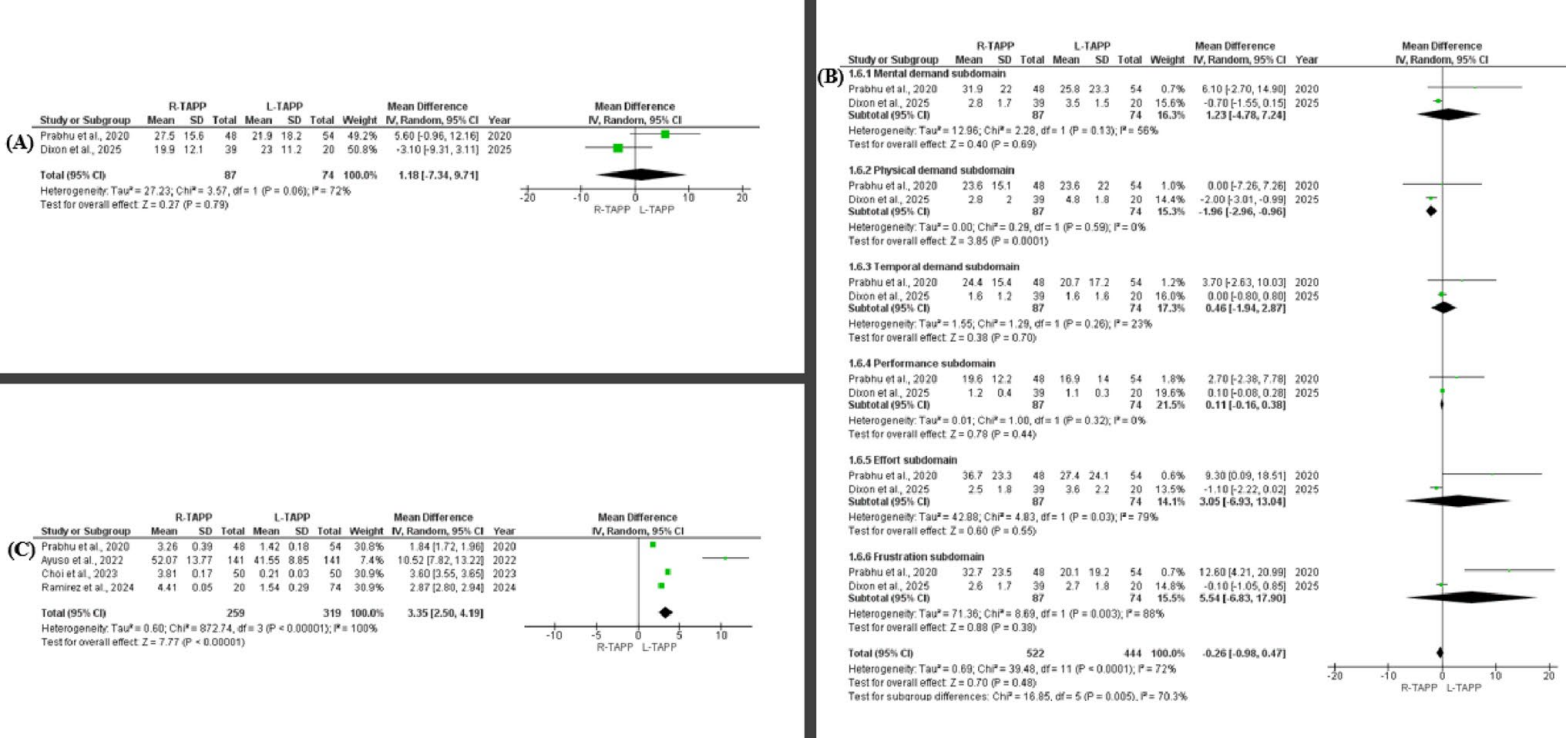
Forest plots comparing surgeon workload and procedural cost between robotic and laparoscopic TAPP inguinal hernia repair using random-effects meta-analysis. **(A)** Total NASA Task Load Index (NASA-TLX) score (mean difference [MD]). **(B)** NASA-TLX subdomain scores (MD), including mental demand, physical demand, temporal demand, performance, effort, and frustration. **(C)** Procedural cost (USD; MD). Effect estimates are presented with 95% confidence intervals (CIs); heterogeneity is reported using the I² statistic

Overall workload, as measured by the total NASA Task Load Index, was reported in two studies [34, 36], comprising 87 robotic procedures and 74 laparoscopic procedures. Pooled analysis demonstrated no significant difference in total workload scores between R-TAPP and L-TAPP (MD 1.18; 95% CI − 7.34 to 9.71; *p* = 0.79), although substantial heterogeneity was observed (I² = 72%) (Fig. 5A). The observed inconsistency between studies is likely attributable to methodological differences in workload assessment. Specifically, one study employed the original NASA-TLX instrument [36], whereas the other utilized a modified scoring format [34], potentially contributing to variability in pooled estimates.

#### Surgeon workload and ergonomics: NASA task load index scale

Two studies [34, 36] provided data on surgeon-reported workload using the Operating Surgeon NASA Task Load Index. Across these studies, 522 procedures were performed using the robotic approach and 444 using laparoscopy. The pooled analysis demonstrated no statistically significant difference in overall NASA-TLX scores between R-TAPP and L-TAPP (MD − 0.28; 95% CI − 0.98 to 0.47; *p* = 0.48), although between-study heterogeneity was considerable (I² = 72%). Subdomain analysis revealed differential patterns across workload components. Mental demand scores did not differ significantly between techniques (MD 1.23; 95% CI − 4.78 to 7.24; *p* = 0.69; I² = 56%). In contrast, the physical demand component showed a statistically significant reduction in favor of the robotic platform (MD − 1.96; 95% CI − 2.96 to − 0.96; *p* = 0.0001), with no observed heterogeneity (I² = 0%). Temporal demand demonstrated no significant difference (MD 0.46; 95% CI − 1.94 to 2.87; *p* = 0.70; I² = 23%). Similarly, performance (MD 0.11; 95% CI − 0.16 to 0.38; *p* = 0.44; I² = 0%), effort (MD 3.05; 95% CI − 6.93 to 13.04; *p* = 0.55; I² = 79%), and frustration (MD − 0.26; 95% CI − 0.98 to 0.47; *p* = 0.38; I² = 72%) did not reach statistical significance. The overall test for subgroup interaction was statistically significant (*p* = 0.005), accompanied by substantial heterogeneity (I² = 70.3%), suggesting variability in how individual workload dimensions differed between surgical platforms (Fig. 5B).

#### Cost analysis (USD)

Cost data were available from four comparative studies [22, 23, 25, 36], including 259 patients in the robotic cohort and 319 in the laparoscopic cohort. Quantitative synthesis demonstrated a clear economic disparity favoring the laparoscopic approach. On pooled analysis, robotic TAPP incurred an average additional cost of approximately $3,350 per procedure (MD 3.35; 95% CI 2.50–4.19; *p* < 0.00001) (Fig. 5C). Considerable statistical heterogeneity was observed (I² = 100%), suggesting substantial variability across studies. This variability is most plausibly attributable to differences in institutional costing frameworks, capital investment amortization strategies, disposable instrumentation expenses, and operating room time valuation within distinct healthcare systems.

### Publication bias

Assessment of potential small-study effects for the operative time outcome was performed using funnel plot visualization (Figure S3). The distribution of studies demonstrated evident asymmetry, with smaller trials tending to report larger effect estimates favoring one approach. This pattern raises concern for possible publication bias, although alternative explanations—such as between-study variability in surgical expertise, case complexity, or methodological design—cannot be excluded. To complement the visual inspection, Egger’s regression analysis was applied as a statistical test for asymmetry. The test produced a significant result (*p* = 0.028), supporting the presence of a small-study effect.

## Discussion

This updated meta-analysis synthesizing data from 5,520 patients provides a contemporary reassessment of the comparative performance of robotic and laparoscopic TAPP repair. While robotic assistance was associated with increased operative duration and higher procedural expenditure, clinical endpoints—including recurrence, postoperative morbidity, and chronic inguinodynia—remained statistically comparable between approaches. The principal differentiator emerged in surgeon-centered ergonomics, where robotic platforms demonstrated reduced physical workload. Heterogeneity was pronounced across included studies for several outcomes, particularly operative time and cost, reflecting variation in surgical protocols, institutional practices, and surgeon experience level. Operative time comparisons may be affected by selection bias, as robotic platforms are sometimes used for more complex cases. Thus, the longer durations seen in robotic cohorts may indicate case complexity rather than a technological limitation, contributing to observed variability.

Our finding that the R-TAPP group has longer operative time aligns with the majority of published meta-analyses. Li et al. reported a mean difference of 14.02 min favoring L-TAPP [38]. Similarly, Qabbani et al.‘s analysis of 19 studies with 8987 patients reported longer robotic operative times [39]. However, a recent randomized controlled trial (DIRECT) demonstrated that R-TAPP was 9 min faster than L-TAPP for simple hernias (51 vs. 60 min) and 20 min faster for complex hernias (72 vs. 92 min), even after accounting for the 5-minute robotic docking time [33]. This discrepancy likely reflects the influence of surgeon experience and learning curve on robotic operative efficacy. For instance, studies published earlier between 2016 and 2020 predominantly captured surgeons during their early adoption phase, when proficiency had not yet been achieved [32]. The high heterogeneity (I² = 95%) in our operative time analysis supports this explanation, as the included studies reflect different phases of institutional robotic experience. Muysoms et al. examined the learning curve and reported a notable decrease in operative time with increasing case volume [27]. More recent data from experienced centers performing > 300 robotic cases show comparable or even shorter operative times compared to laparoscopic repairs [40]. Notably, the operative time disadvantage of R-TAPP diminishes substantially after the learning curve is overcome [41, 42]. Furthermore, studies demonstrated that approximately 20–43 cases are required to achieve proficiency, with remarkable improvement in operative time and complication rates [37, 43]. The interpretation of operative time differences highlights important directions for future research. Upcoming comparative studies should adopt standardized reporting of operative duration by explicitly separating docking, operative (console/dissection), and closure/undocking phases, as aggregation of these components may obscure true procedural efficiency. Operative time analyses should also be stratified by hernia complexity, surgeon experience, and learning-curve stage, given their substantial influence on workflow and efficiency. Prospective randomized or multicenter studies are encouraged to prespecify operative time as a secondary endpoint and to report robotic platform generation and team familiarity. Such methodological standardization is essential to distinguish transient adoption-related delays from genuine platform-specific performance characteristics.

Chronic postoperative inguinal pain (inguinodynia) is a fundamental patient-centered outcome following hernia repair. The outcome was reported in three of the included studies, yet our analysis found no significant difference between R-TAPP and L-TAPP. Despite contradicting the theoretical advantage often attributed to robotic procedures, our finding echoes the ROLAIS trial, which reported similar chronic pain rates at 3-month follow-up [31]. The absence of a significant difference may reflect the shared transabdominal approach of both techniques, which theoretically minimizes direct nerve handling compared to open repair [42]. However, our findings should be interpreted cautiously due to the limited number of studies (*n* = 3) and relatively short follow-up duration (median 3–6 months). Additionally, the lack of standardized pain assessment tools across included studies introduces measurement heterogeneity that may preclude subtle differences between the two techniques. Furthermore, definitions of chronic postoperative pain varied between studies, with some investigations relying on patient-reported pain scales while others defined inguinodynia based on clinical follow-up documentation. This variability in outcome measurement introduces methodological heterogeneity that may limit the ability to detect small differences between surgical platforms.

The remarkably higher costs of R-TAPP represent a critical barrier to widespread adoption and warrant careful consideration within the context of healthcare utilization. Our findings align with Li et al., who reported a $4170 USD cost difference [38], and Hinojosa-Ramirez et al., who reported total hospitalization costs of €3272 for R-TAPP vs. €1049 for L-TAPP [25]. Furthermore, these cost gaps primarily stem from three sources: robotic capital expenditures, disposable instrument costs, and extended operating room occupancy, particularly during the learning phase. However, cost-effective analysis must account for indirect savings from reduced chronic pain rates, faster return to work, and decreased long-term health care utilization [44, 45]. Notably, these outcomes become apparent with extended follow-up. The high heterogeneity (I² = 100%) observed in our cost analysis reflects substantial variations in institutional pricing plans, reimbursement models, and accounting methodologies. These factors together limit the generalizability of absolute cost figures across different healthcare systems.

Our meta-analysis demonstrated no significant difference in overall complication rates, seroma formation, hematomas, surgical site infections (SSIs), or recurrence rates between R-TAPP and L-TAPP. Conversion to open repair is a key indicator of intraoperative difficulties with minimally invasive techniques. In this review, conversion events were rare, with only one case in the laparoscopic cohort and none in the robotic cohort. However, the limited number of events and inconsistent reporting hinder definitive conclusions about differences between platforms. Notably, exploratory subgroup analysis stratified by dominant mesh type suggested a potential interaction between surgical platform and seroma formation. In studies utilizing self-fixating meshes, robotic TAPP was associated with a significantly lower risk of seroma compared with laparoscopic TAPP, whereas no difference was observed in studies using polypropylene or contoured meshes. This finding should be interpreted cautiously and considered hypothesis-generating rather than confirmatory. The observed effect may reflect interactions between mesh characteristics, fixation strategy, and defect closure practices, features that are more consistently standardized and ergonomically facilitated in robotic repair, rather than a direct platform-specific advantage.

Given the borderline statistical significance of the subgroup interaction and the limited number of contributing studies, these results should not be used to alter clinical practice at this stage and warrant confirmation in adequately powered randomized trials with standardized mesh and fixation protocols.

In the broader context of postoperative safety, These findings diverge from a recent meta-analysis by Khewater et al. that reported a threefold increased risk of SSIs with robotic repair. This discrepancy may be attributable to differences in study inclusion criteria, with our analysis focusing exclusively on TAPP-to-TAPP comparisons instead of pooling diverse approaches that may have included the totally extraperitoneal (TEP) technique. In addition, the comparable safety profile observed in our study is further supported by a recent cohort study that revealed similar rates of postoperative complications among the two groups [31]. Analysis of complications stratified by Clavien-Dindo classification revealed no significant differences across minor (Grade I-II) or major (Grade III) complications, suggesting that R-TAPP doesn’t substantially alter the risk profile when performed by an adequately trained surgeon. Furthermore, long-term recurrence data extending to 8 years from single-institution cohorts reported remarkable low rates of 0.46% for R-TAPP, comparable to best-practice laparoscopic outcomes [40].

Blood loss was statistically equivalent between R-TAPP and L-TAPP, indicating comparable hemostatic control and tissue handling precision during hernia sac manipulation and preperitoneal dissection. These findings also reflect the protective hemostatic effects of pneumoperitoneum-induced tamponade and meticulous tissue dissection inherent to both minimally invasive techniques. However, emerging surgical stress response biomarkers reveal subtle physiologic differences. For instance, the ROLAIS randomized controlled trial reported significantly lower C-reactive protein and IL-6 in R-TAPP, suggesting that enhanced robotic visualization, articulation, and tissue handling precision manifest as reduced systemic inflammatory response [37]. Subsequently, this may lead to accelerated recovery and reduced postoperative pain. However, we reported no significant difference regarding the length of hospital stay between the two approaches.

In addition, an important clinically relevant finding from our analysis was a significant reduction in physical demand experienced by surgeons performing R-TAPP as measured by the NASA task load index. Dixon et al., in the VOLTAIRE trial, specifically designed to assess ergonomic outcomes, showed that robotic assistance substantially reduced surgeon musculoskeletal strain, particularly during complex bilateral repairs and prolonged procedures [34]. This finding addresses a critical but often overlooked dimension of surgical quality: the sustainability of surgical practice over a surgeon’s career. Laparoscopic surgery, while superior to open approaches in patient outcomes, imposes significant ergonomic strain due to fixed instrument angles, counterintuitive hand-eye coordination, and prolonged static positioning [46]. On the other hand, the robotic platform’s articulating instruments, tremor filtration, and ergonomic console design may translate to reduced occupational injury rates and prolonged surgeon productivity [47]. Nevertheless, the benefits are difficult to quantify in traditional cost-effective analyses.

There was no statistically significant difference regarding urinary complications, such as postoperative urinary retention (POUT) and urinary tract infections (UTIs), between the two groups, which aligns with a recent randomized clinical trial by Prabhu et al. [36]. Furthermore, RETAINER I, a multicenter study of 4151 patients, identified significant POUR risk factors such as advanced age, anticholinergic medication use, history of urinary retention, constipation, prolonged operative time, and bladder involvement within the hernia—factors independent of the surgical platform [48].

This systematic review and meta-analysis advances the evidence base beyond prior work through several methodological refinements. First, unlike Li et al. [38], which evaluated 10 studies, the current work incorporates data from two recent RCTs (ROLAIS [37] and VOLTAIRE [34]) Plus three cohort studies published in 2024, expanding the sample size to 5520 patients. In addition, the comprehensive assessment of both primary outcomes (operative time, chronic pain) and 15 secondary endpoints, such as NASA-TLX scores, Clavien-Dindo classification, and cost analysis, provides a more granular evaluation of surgical efficacy and safety than prior studies.

On the other hand, our study has several important limitations. First, the substantial heterogeneity observed across multiple outcomes (I² ranging from 52% to 100%) reflects remarkable variability in surgical techniques, mesh types, fixation methods, defect closure protocols, and follow-up durations among the included studies. Second, the majority of included studies were observational cohort designs with only three RCTs, introducing potential selection bias and confounding by indication; additionally, surgeons may have preferentially assigned more complex cases to one platform over another [49]. Only three randomized controlled trials were included, while most evidence came from observational cohort studies. Therefore, potential confounding and differences in case selection between robotic and laparoscopic approaches remain possible. Third, the relatively short follow-up period (median 1–12 months) is insufficient to capture long-term outcomes such as late recurrence and chronic neuropathic pain, which may not become evident until 2–5 years postoperatively [50, 51]. Finally, the learning curve effect is a fundamental confounding variable that was not systematically controlled across studies; many publications from 2016 to 2020 likely captured surgeons during their initial robotic cases. These limitations should be considered when interpreting the pooled estimates and highlight the need for large multicenter randomized trials with standardized outcome definitions.

Emerging technologies are expected to reshape the surgical landscape for inguinal hernia repair. For instance, a recent learning-curve study of the Da Vinci Single Port platform reported transferable skills from multiport systems and a reduction in median operative time from 82 to 62 min after minimal experience [52]. Additionally, AI integration through machine learning models can now predict surgical complexity requiring component separation with 81.3% accuracy compared with 65% for expert surgeons and estimate SSI risk with an AUC of 0.898 [53].

## Conclusion

Current evidence suggests that robotic TAPP may be associated with longer operative duration and higher procedural costs compared with laparoscopic TAPP, while achieving comparable short-term clinical outcomes in terms of safety and effectiveness. Therefore, the selection of surgical approach should be individualized, guided by surgeon proficiency, institutional capabilities, hernia complexity, and patient-specific considerations. The most consistent advantage of the robotic platform appears to be improved surgeon ergonomics, particularly reduced physical strain, with emerging signals of potential benefit in technically challenging or complex hernias, especially in high-volume centers that have progressed beyond the learning curve. As advancements such as single-port robotic systems and artificial intelligence–enhanced surgical planning continue to evolve, the role of robotic repair may broaden. Nonetheless, laparoscopic TAPP is likely to remain the more economically sustainable option for routine inguinal hernia repair, particularly in healthcare systems with limited resources. However, these findings should be interpreted cautiously, given the predominance of observational evidence and the heterogeneity across studies. Future large-scale, methodologically rigorous randomized trials with extended follow-up are needed to better define the long-term comparative value of these approaches.

## Supplementary Information

Below is the link to the electronic supplementary material.

Supplementary Material 1

Supplementary Material 2

Supplementary Material 3

## Data Availability

This published article and its supplementary materials include all data generated or analyzed during this study.

## Abbreviations

CDVTI: Cohort De-duplication and Verification of Trial Independence
I²: I-squared statistic
MD: Mean difference
NR: Not reported
PCS: Prospective cohort study
PCCS: Prospective case–control study
RCS: Retrospective cohort study
RR: Risk ratio
ROBINS-I: Risk Of Bias In Non-randomized Studies of Interventions
RoB 2: Risk of Bias 2 tool
NASA-TLX: National Aeronautics and Space Administration Task Load Index
POUT: Postoperative urinary retention
