# Supplementary material for: Short-term outcomes of robotic versus laparoscopic TAPP for inguinal hernia repair: a systematic review, meta-analysis, and GRADE assessment
Source: J Robot Surg. 2026 Apr 6;20(1):419. doi: 10.1007/s11701-026-03335-3 (PMC13053352; doi:10.1007/s11701-026-03335-3)
Supplement: Supplementary file 1 — Supplementary Material 1 [file 11701_2026_3335_MOESM1_ESM.docx]

**Table 1. Certainty of evidence and clinical outcomes for robotic-assisted versus laparoscopic TAPP inguinal hernia repair.**

**Author(s):**

**Question:** Robotic-assisted transabdominal preperitoneal inguinal hernia repair compared to laparoscopic transabdominal preperitoneal inguinal hernia repair

**Setting:**

**Bibliography:** . robotic-assisted transabdominal preperitoneal inguinal hernia repair versus laparoscopic transabdominal preperitoneal inguinal hernia repair for [health problem]. Cochrane Database of Systematic Reviews [Year], Issue [Issue].

| **Certainty assessment** | | | | | | | **№ of patients** | | **Effect** | | **Certainty** | **Importance** |
| --- | --- | --- | --- | --- | --- | --- | --- | --- | --- | --- | --- | --- |
| **№ of studies** | **Study design** | **Risk of bias** | **Inconsistency** | **Indirectness** | **Imprecision** | **Other considerations** | **robotic-assisted transabdominal preperitoneal inguinal hernia repair** | **laparoscopic transabdominal preperitoneal inguinal hernia repair for [health problem** | **Relative (95% CI)** | **Absolute (95% CI)** |  |  |
| **Operative time (min)** | | | | | | | | | | | | |
| 12 | non-randomised studies | serious^a^ | very serious^b^ | not serious | not serious | publication bias strongly suspected very strong association^c^ | 2582 | 2499 | - | MD **10.93 higher** (4.18 higher to 17.67 higher) | ⨁⨁◯◯ Low^a,b,c^ | CRITICAL |
| **Blood loss (ml)** | | | | | | | | | | | | |
| 3 | non-randomised studies | not serious | not serious | not serious | serious^d^ | strong association | 295 | 286 | - | MD **0.32 lower** (2.15 lower to 1.52 higher) | ⨁⨁⨁◯ Moderate^a,d^ | CRITICAL |
| **Length of hospital stay (days)** | | | | | | | | | | | | |
| 5 | non-randomised studies | serious^a^ | not serious | not serious | serious^d^ | strong association | 197 | 278 | - | MD **0.29 higher** (0.1 higher to 0.49 higher) | ⨁⨁⨁◯ Moderate^a,d^ | CRITICAL |
| **Total NASA-TLX** | | | | | | | | | | | | |
| 2 | randomised trials | serious^a^ | serious^e^ | not serious | serious^d^ | strong association | 87 | 74 | - | MD **1.18 higher** (7.34 lower to 9.71 higher) | ⨁⨁◯◯ Low^a,d,e^ | IMPORTANT |
| **Operating Surgeon NASA Task Load Index Scale** | | | | | | | | | | | | |
| 2 | randomised trials | serious^a^ | not serious | not serious | not serious | none | 522 | 444 | - | MD **0.26 lower** (0.98 lower to 0.47 higher) | ⨁⨁⨁◯ Moderate^a^ | IMPORTANT |
| **Seroma** | | | | | | | | | | | | |
| 8 | non-randomised studies | serious^a^ | not serious | not serious | not serious | none | 91/2080 (4.4%) | 177/2131 (8.3%) | **RR 0.73** (0.43 to 1.24) | **22 fewer per 1,000** (from 47 fewer to 20 more) | ⨁⨁⨁◯ Moderate^a^ | CRITICAL |
| **Haematoma** | | | | | | | | | | | | |
| 7 | non-randomised studies | not serious | not serious | not serious | not serious | none | 27/2057 (1.3%) | 34/2053 (1.7%) | **RR 0.80** (0.47 to 1.36) | **3 fewer per 1,000** (from 9 fewer to 6 more) | ⨁⨁⨁⨁ High | CRITICAL |
| **Recurrence** | | | | | | | | | | | | |
| 8 | non-randomised studies | not serious | not serious | not serious | not serious | none | 13/2229 (0.6%) | 22/2239 (1.0%) | **RR 0.61** (0.31 to 1.19) | **4 fewer per 1,000** (from 7 fewer to 2 more) | ⨁⨁⨁◯ Moderate^a^ | IMPORTANT |
| **Readmission** | | | | | | | | | | | | |
| 7 | non-randomised studies | serious^a^ | not serious | not serious | not serious | none | 26/2324 (1.1%) | 36/2228 (1.6%) | **RR 0.74** (0.34 to 1.61) | **4 fewer per 1,000** (from 11 fewer to 10 more) | ⨁⨁⨁◯ Moderate^a^ | IMPORTANT |
| **Urinary retention** | | | | | | | | | | | | |
| 5 | non-randomised studies | serious^a^ | not serious | not serious | not serious | none | 30/717 (4.2%) | 34/582 (5.8%) | **RR 0.74** (0.46 to 1.21) | **15 fewer per 1,000** (from 32 fewer to 12 more) | ⨁⨁⨁◯ Moderate^a^ | IMPORTANT |
| **Overall complication rate** | | | | | | | | | | | | |
| 8 | non-randomised studies | very serious^f^ | not serious | not serious | not serious | none | 177/2281 (7.8%) | 264/2181 (12.1%) | **RR 0.85** (0.59 to 1.24) | **18 fewer per 1,000** (from 50 fewer to 29 more) | ⨁⨁◯◯ Low^f^ | CRITICAL |
| **Complications according to Clavien-Dindo classification** | | | | | | | | | | | | |
| 5 | non-randomised studies | serious^a^ | not serious | not serious | not serious | none | 40/1444 (2.8%) | 50/1113 (4.5%) | **RR 0.98** (0.78 to 1.24) | **1 fewer per 1,000** (from 10 fewer to 11 more) | ⨁⨁⨁◯ Moderate^a^ | CRITICAL |
| **Cost (USD)** | | | | | | | | | | | | |
| 4 | non-randomised studies | serious^a^ | not serious | not serious | serious^d^ | very strong association | 259 | 319 | - | MD **3.35 higher** (2.5 higher to 4.19 higher) | ⨁⨁⨁◯ Moderate^a^ | IMPORTANT |
| **Reoperation** | | | | | | | | | | | | |
| 2 | non-randomised studies | serious^a^ | not serious | not serious | serious^d^ | none | 4/1618 (0.2%) | 7/1676 (0.4%) | **RR 0.73** (0.22 to 2.35) | **1 fewer per 1,000** (from 3 fewer to 6 more) | ⨁⨁◯◯ Low^a,d^ | IMPORTANT |
| **Chronic pain (Inguinodynia)** | | | | | | | | | | | | |
| 3 | non-randomised studies | not serious | not serious | not serious | serious^d^ | strong association | 13/308 (4.2%) | 28/308 (9.1%) | **RR 0.58** (0.30 to 1.12) | **38 fewer per 1,000** (from 64 fewer to 11 more) | ⨁⨁⨁◯ Moderate^a^ | IMPORTANT |
| **Surgical site infection** | | | | | | | | | | | | |
| 4 | non-randomised studies | serious^a^ | not serious | not serious | not serious | none | 4/1706 (0.2%) | 8/1810 (0.4%) | **RR 0.75** (0.25 to 2.27) | **1 fewer per 1,000** (from 3 fewer to 6 more) | ⨁⨁⨁◯ Moderate^a^ | IMPORTANT |
| **Urinary tract infection** | | | | | | | | | | | | |
| 2 | non-randomised studies | not serious | not serious | not serious | not serious | none | 2/1672 (0.1%) | 3/1663 (0.2%) | **RR 0.68** (0.10 to 4.72) | **1 fewer per 1,000** (from 2 fewer to 7 more) | ⨁⨁⨁◯ Moderate^a^ | IMPORTANT |

**CI:** confidence interval; **MD:** mean difference; **RR:** risk ratio

#### Explanations

a. Some concerns in the included Studies

b. High heterogeneity in the included studies

c. The funnel plot used to assess publication bias showed a notable level of asymmetry.

d. Small sample size and included studies.

e. Moderate heterogeneity in the included studies

f. High risk of bias and some concerns in most included Studies
