# Supplementary material for: Short-term outcomes of robotic versus laparoscopic TAPP for inguinal hernia repair: a systematic review, meta-analysis, and GRADE assessment
Source: J Robot Surg. 2026 Apr 6;20(1):419. doi: 10.1007/s11701-026-03335-3 (PMC13053352; doi:10.1007/s11701-026-03335-3)
Supplement: Supplementary file 2 — Supplementary Material 2 [file 11701_2026_3335_MOESM2_ESM.docx]

**Supplementary File 1**

**Search Strategy Used for the Systematic Review (Executed on September 12, 2025)**

A search strategy was employed across five databases—PubMed, Scopus, Web of Science, Cochrane Library, and Embase—to identify studies comparing Robotic TAPP with Laparoscopic TAPP. The search was designed using combinations of Medical Subject Headings (MeSH) and relevant free-text terms.

**PubMed**

("Hernia, Inguinal"[Mesh] OR "Inguinal Hernia" OR "groin hernia" OR "inguinal herni*") AND (("Robotics"[Mesh] OR "Robotic Surgical Procedures"[Mesh] OR robot* OR "robot-assisted" OR "robotic-assisted" OR "da Vinci" OR davinci OR "robotic TAPP" OR "robotic transabdominal preperitoneal" OR "robotic transabdominal pre-peritoneal") OR ("minimally invasive robotic")) AND (("Laparoscopy"[Mesh] OR laparoscopy OR laparoscop* OR "laparoscopic TAPP" OR "transabdominal preperitoneal" OR "TAPP" OR "trans-abdominal preperitoneal"))

**Scopus**

TITLE-ABS-KEY ("Hernia, Inguinal" OR "Inguinal Hernia" OR "groin hernia" OR "inguinal herni*") AND TITLE-ABS-KEY (("Robotics" OR "Robotic Surgical Procedures" OR robot* OR "robot-assisted" OR "robotic-assisted" OR "da Vinci" OR davinci OR "robotic TAPP" OR "robotic transabdominal preperitoneal" OR "robotic transabdominal pre-peritoneal") OR ("minimally invasive robotic")) AND TITLE-ABS-KEY (("Laparoscopy" OR laparoscopy OR laparoscop* OR "laparoscopic TAPP" OR "transabdominal preperitoneal" OR "TAPP" OR "trans-abdominal preperitoneal"))

**Web of Science**

TOPIC: ("Hernia, Inguinal" OR "Inguinal Hernia" OR "groin hernia" OR "inguinal herni*") AND TOPIC: (("Robotics" OR "Robotic Surgical Procedures" OR robot* OR "robot-assisted" OR "robotic-assisted" OR "da Vinci" OR davinci OR "robotic TAPP" OR "robotic transabdominal preperitoneal" OR "robotic transabdominal pre-peritoneal") OR ("minimally invasive robotic")) AND TOPIC: (("Laparoscopy" OR laparoscopy OR laparoscop* OR "laparoscopic TAPP" OR "transabdominal preperitoneal" OR "TAPP" OR "trans-abdominal preperitoneal"))

**Cochrane**

("Hernia, Inguinal"[Mesh] OR "Inguinal Hernia" OR "groin hernia" OR "inguinal herni*") AND (("Robotics"[Mesh] OR "Robotic Surgical Procedures"[Mesh] OR robot* OR "robot-assisted" OR "robotic-assisted" OR "da Vinci" OR davinci OR "robotic TAPP" OR "robotic transabdominal preperitoneal" OR "robotic transabdominal pre-peritoneal") OR ("minimally invasive robotic")) AND (("Laparoscopy"[Mesh] OR laparoscopy OR laparoscop* OR "laparoscopic TAPP" OR "transabdominal preperitoneal" OR "TAPP" OR "trans-abdominal preperitoneal"))

**Embase**

("Hernia, Inguinal" OR "Inguinal Hernia" OR "groin hernia" OR "inguinal herni*") AND (("Robotics"[Mesh] OR "Robotic Surgical Procedures" OR robot* OR "robot-assisted" OR "robotic-assisted" OR "da Vinci" OR davinci OR "robotic TAPP" OR "robotic transabdominal preperitoneal" OR "robotic transabdominal pre-peritoneal") OR ("minimally invasive robotic")) AND (("Laparoscopy" OR laparoscopy OR laparoscop* OR "laparoscopic TAPP" OR "transabdominal preperitoneal" OR "TAPP" OR "trans-abdominal preperitoneal"))

**ClinicalTrials.gov**

(inguinal hernia) AND (TAPP OR "transabdominal preperitoneal") AND (robotic OR "robot-assisted" OR "da Vinci") AND (laparoscop* OR conventional) AND (inguinodynia OR "chronic pain" OR "postoperative pain" OR "groin pain" OR "operative time" OR cost OR recurrence)
