# Supplementary material for: Short-term outcomes of robotic versus laparoscopic TAPP for inguinal hernia repair: a systematic review, meta-analysis, and GRADE assessment
Source: J Robot Surg. 2026 Apr 6;20(1):419. doi: 10.1007/s11701-026-03335-3 (PMC13053352; doi:10.1007/s11701-026-03335-3)
Supplement: Supplementary file 3 — Supplementary Material 3 [file 11701_2026_3335_MOESM3_ESM.docx]

**Supplementary File 2**

**CDVTI Flagged Studies**

**Table S1. Studies Flagged by CDVTI and Inclusion Decisions**

| **CDVTI Flagged Pair** | **Primary Study (Included)** | **Basis for CDVTI Flag** | **Inclusion Decision Rationale** |
| --- | --- | --- | --- |
| Prabhu et al., 2020 vs Miller et al., 2023 | Prabhu et al., 2020 | Overlapping patient cohort (RIVAL trial), identical intervention arms (laparoscopic vs robotic TAPP), shared investigators, and continuation of follow-up | Prabhu et al. (2020) was included as the index randomized trial reporting primary short-term outcomes. Miller et al. (2023) represents a 2-year follow-up analysis of the same cohort and was excluded to avoid double-counting participants |
| Valorenzos et al., 2025 vs Arunthavanathan et al., 2025 | Valorenzos et al., 2025 | Identical randomized cohort (Danish RCT), overlapping enrolment period, same institution, identical intervention comparison (R-TAPP vs L-TAPP) | Valorenzos et al. (2025) was retained as the primary trial report with prespecified outcomes and full methodological transparency. Arunthavanathan et al. (2025) was excluded as a derivative analysis of the same dataset |

**Abbreviations:** CDVTI, Cross-Dataset Verification and Trial Integrity; TAPP, transabdominal preperitoneal repair.

**Table S2. Network Authorship Assessment Across Included Studies**

| **Study** | **Authorship Overlap with Other Included Studies** | **Institutional Overlap** | **Network Handling Decision** | **Rationale** |
| --- | --- | --- | --- | --- |
| Prabhu et al., 2020 | Yes (shared authors with Miller et al., 2023) | Yes (Cleveland Clinic and collaborating centers) | Included once; duplicate cohort excluded | Authorship overlap reflected longitudinal reporting of the same randomized cohort rather than independent evidence  Prabhu et al., 2020 |
| Valorenzos et al., 2025 | Yes (shared investigators with Arunthavanathan et al., 2025) | Yes (University Hospital of Southern Denmark network) | Included once; derivative study excluded | Network authorship confirmed dataset reuse; inclusion restricted to the most comprehensive trial report |

**Application of CDVTI to Included Studies**

Cross-Dataset Verification and Trial Integrity (CDVTI) was applied to identify overlapping study populations and prevent double counting of participants across publications. When multiple reports were identified from the same randomized cohort, only a single study was retained based on prespecified criteria prioritizing the index randomized trial or the most methodologically comprehensive primary report. Specifically, between Prabhu et al. (2020) and Miller et al. (2023), Prabhu et al. was included as the original randomized trial, while Miller et al. was excluded as a longer-term follow-up analysis of the same cohort. Similarly, between Valorenzos et al. (2025) and Arunthavanathan et al. (2025), Valorenzos et al. was retained as the primary randomized trial report, and Arunthavanathan et al. was excluded as a derivative analysis based on the same underlying dataset. These decisions were made a priori to ensure that each participant contributed only once to the evidence base and that pooled estimates reflected independent observations. Assessment of network authorship and investigator overlap was conducted separately and is reported independently from CDVTI determinations.

**
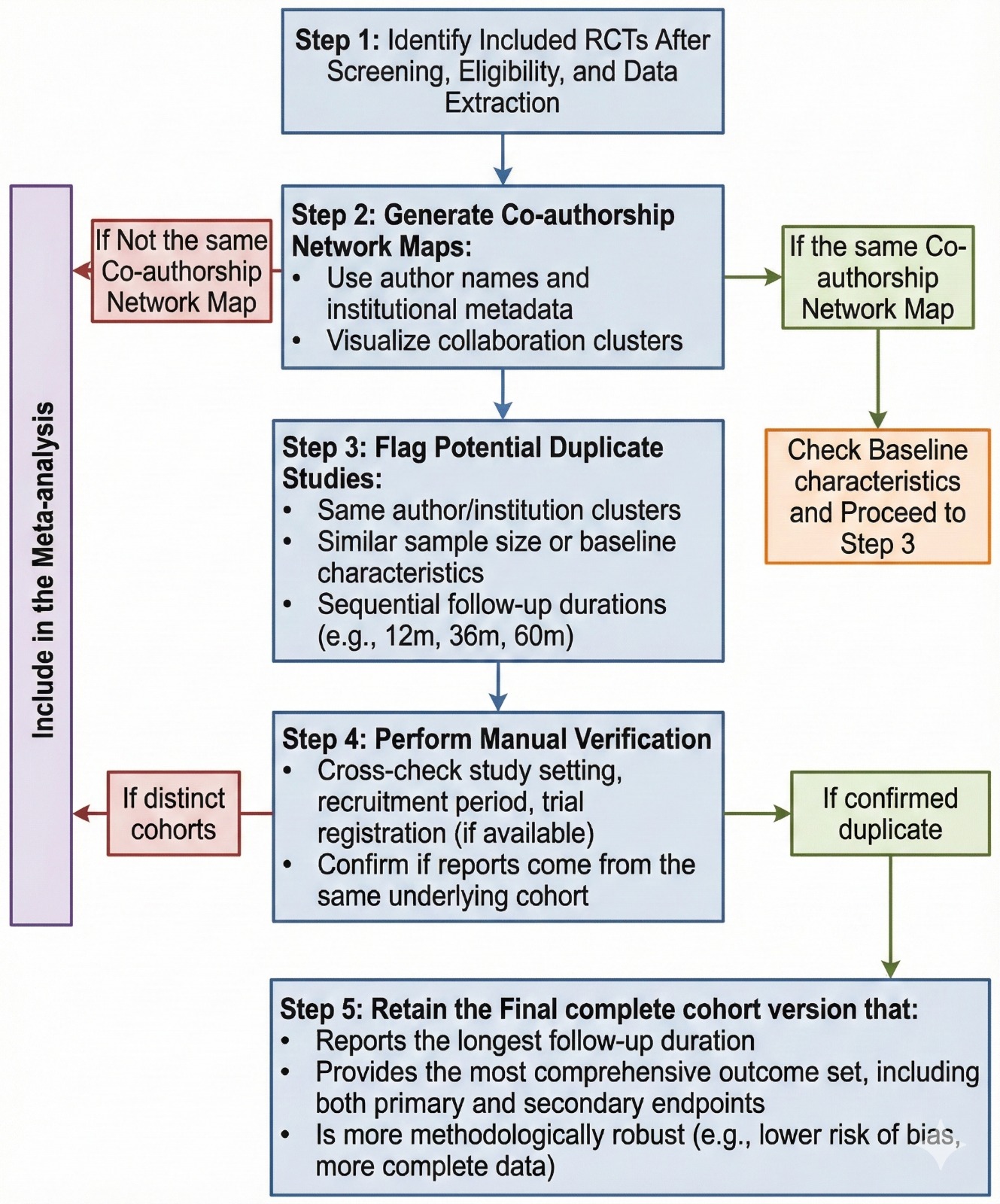
**

**Figure S1.** Schematic representation of the Cross-Dataset Verification and Trial Integrity (CDVTI) workflow used to identify overlapping randomized trial cohorts and retain a single independent dataset for meta-analysis.


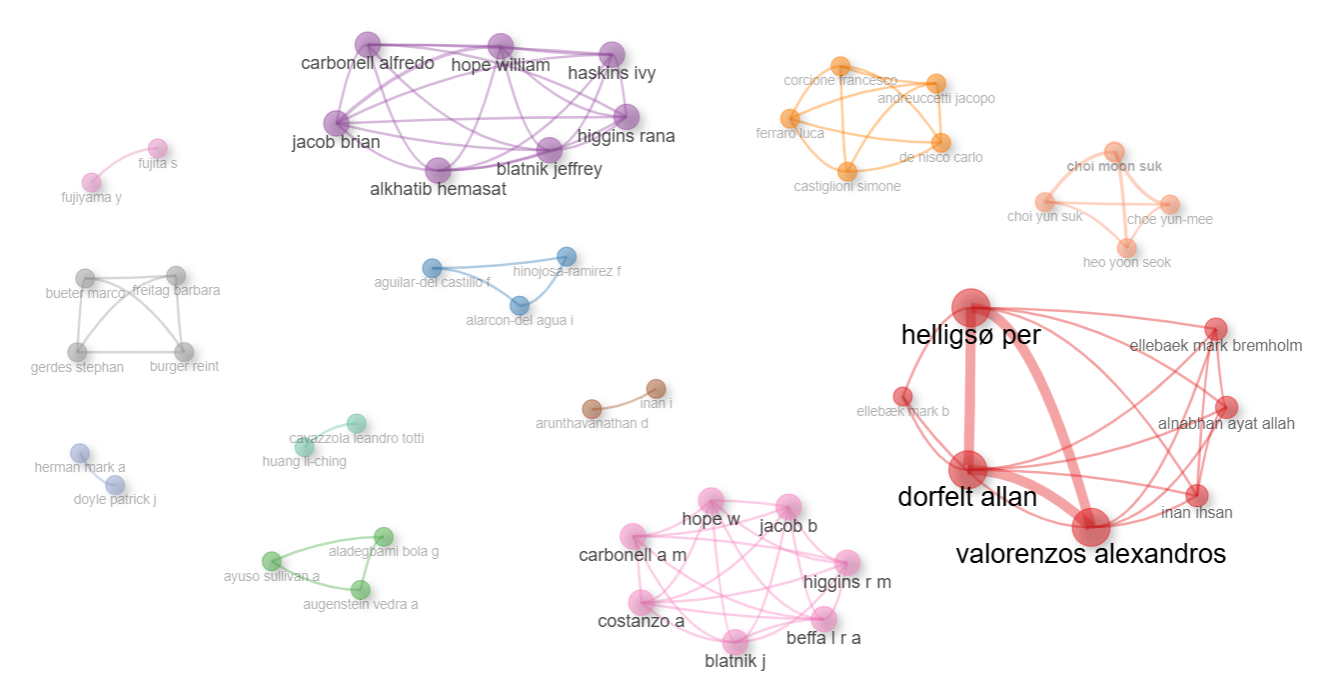


**Figure S2.** Authorship network visualization of included studies comparing robotic and laparoscopic transabdominal preperitoneal (TAPP) inguinal hernia repair, illustrating co-authorship relationships and investigator overlap used to support Cross-Dataset Verification and Trial Integrity (CDVTI) adjudication.


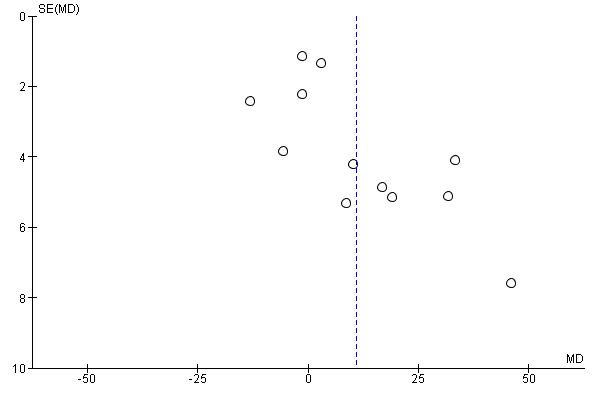


**Figure S3.** Funnel plot assessing small-study effects and potential publication bias for operative time (minutes) comparing robotic versus laparoscopic transabdominal preperitoneal (TAPP) inguinal hernia repair. The x-axis represents the mean difference (MD) in operative time, and the y-axis represents the standard error of the mean difference [SE(MD)]. The dashed vertical line indicates the pooled effect estimate derived from the random-effects meta-analysis.
